# Osteosarcoma 3D patient derived cultures to test genome-informed personalized treatment options: a feasibility study

**DOI:** 10.3389/fmed.2026.1754270

**Published:** 2026-03-17

**Authors:** Ieva Palubeckaitė, Sanne Venneker, Natasja Franceschini, Pauline Wijers-Koster, Cedrick Agaser, Inge Briaire-de Bruijn, Alwine B. Kruisselbrink, Brendy van den Akker, Hailiang Mei, Anne-Marie Cleton-Jansen, Hans Gelderblom, Michiel A. J. van de Sande, Judith V. M. G. Bovée

**Affiliations:** 1 Department of Pathology, Leiden University Medical Center, Leiden, Netherlands; 2 Center for Proteomics and Metabolomics, Leiden University Medical Center, Leiden, Netherlands; 3 Sequencing Analysis Support Core, Leiden University Medical Center, Leiden, Netherlands; 4 Department of Medical Oncology, Leiden University Medical Center, Leiden, Netherlands; 5 Department of Orthopaedic Surgery, Leiden University Medical Center, Leiden, Netherlands

**Keywords:** 3D *in vitro* models, osteosarcoma, patient-derived organoids, personalized treatment, tumoroids, WES

## Abstract

**Background:**

Improving osteosarcoma treatment beyond conventional (neo)adjuvant chemotherapy and resection remains challenging. An urgent need for novel therapeutic options, particularly personalized and targeted approaches, has emerged due to high inter-patient molecular heterogeneity. A lack of representative *in vitro* and *in vivo* models impedes therapeutic development, therefore we aimed to create 3D *in vitro* long term culture models directly from patient material.

**Methods:**

Tumour cells from seven osteosarcoma patients were propagated in monolayer or collagen hydrogels, while whole-exome sequencing of corresponding primary tumour tissue was performed to identify potential drug targets. Established cultures were subsequently used to assess efficacy of the identified personalized treatment options.

**Results:**

Three out of seven hydrogel cultures harbored the same genetic alterations as the corresponding primary tumours, but only one culture (L6565) showed viable cells after cryopreservation in combination with long term expansion. Our findings demonstrate feasibility of establishing long-term patient-derived osteosarcoma cultures with a success rate of 14%. This single patient line was used to evaluate genome-informed therapy and to compare cell culture models of increasing complexity. L6565 exhibited homozygous CDKN2A loss with retained Rb expression, rendering tumour cells sensitive to CDK4/CDK6 inhibition via palbociclib. Tumour heterogeneity was reflected in advanced culture methods producing more variability in treatment response.

**Conclusion:**

These results highlight the potential of genome-informed therapies in osteosarcoma and the importance of refining culture techniques to enhance translational research and therapeutic outcomes.

## Introduction

Osteosarcoma, the most common malignant bone sarcoma, is most often diagnosed in children and young adults (<30 years), but also has a second peak of incidence in the elderly (1). Osteosarcoma patient five-year survival rates are as low as 30% in cases of recurrence or relapse. Treatment strategies have not significantly improved survival rates over the last decades. Current treatment consists of highly toxic neoadjuvant methotrexate-doxorubicin-cisplatin (MAP) treatment and surgical resection as no new, improved treatment options have been identified (2, 3). An improved multimodal treatment strategy is critical to improve outcomes for these patients.

The rarity of recurring alterations in combination with the genomic complexity and inter-tumour heterogeneity of osteosarcoma poses a challenge to overcome for future identification of novel treatment options and the development of clinical trials. The potential of clinical genomic profiling of osteosarcoma was demonstrated in both patient cohorts and PDX models (3, 4). Notably, by performing comprehensive analysis of clinically relevant targetable alterations and tailoring treatment strategies in patient-derived xenograft (PDX) models, Sayles and colleagues demonstrated the potential efficacy of genome informed targeted therapy. This approach can be applied to other representative osteosarcoma models, such as 3D cell cultures, in order to gain more information on personalized treatment strategies for the disease.

PDX models are time consuming, labor intensive and require the use of animals. Therefore, an *in vitro* approach to optimize personalized treatment in osteosarcoma would be preferred (5). Conventional 3D cell culture methods involve initial culture of cells in monolayer on a treated plastic surface, with subsequent transfer to low attachment or scaffold-based 3D cultures. This method allows for the evaluation of a treatment response that is more representative of the *in vivo* situation as compared to conventional 2D cell lines (6, 7). *In vitro* models can be further improved by maintaining cells in extracellular matrix-based scaffolds immediately from patient resection material, using methods such as organoid culture (8). In general, sarcoma cells cultured in 2D differ morphologically from the *in vivo* situation due to a lack of cell–cell and cell-matrix interaction, and cells are prone to dedifferentiation over time (9). Continuous monolayer culturing to establish cell lines can select for specific cell populations, alter gene and protein expression, which could be mitigated significantly using 3D culture techniques (10–12). Recapitulating the *in vivo* environment as closely as possible ensures a more representative therapeutic outcome. Organoid-like long-term cultures of sarcomas are still relatively rare, mostly due to difficulties in culturing mesenchymal tumour cells (13). Metastatic osteosarcoma 3D cultures were previously established by adaptation of epithelial organoid culture methods to osteosarcoma samples, however these methods were not effective for long-term primary osteosarcoma material (14). Conversely, short-term patient material culture, without passaging, was reported for osteosarcoma patient material using conventional organoid growth techniques, such as use of Matrigel and defined organoid growth medium (15, 16). The caveat of short term cultures or even tumour explants is that often it is not possible to validate that the cultured cells are tumour cells and not fibroblasts, which is especially challenging in osteosarcoma due to the lack of a specific tumour cell marker.

Long-term stable expansion of osteosarcoma patient lines, unlike immediate use of the cells within the first three passages, allows for continuous use of the model for multiple studies, as a sufficient amount of cells can be cultivated for each study and the osteosarcoma cell fraction can be validated. In the present study we explore the feasibility of generating long-term patient-derived osteosarcoma 3D cell cultures by using methods tailored to the osteosarcoma microenvironment for seven patients. We used whole exome sequencing of the primary tumours of these patients to define genome informed therapies for each patient line. We propagated a selected patient line using both 2D and 3D cell culture approaches. Collagen-based hydrogels and osteogenic growth factors were used for osteosarcoma 3D cell culture propagation straight from patients to prevent 2D culture-based dedifferentiation. Denser cell masses, termed microsarcs, were subsequently produced from hydrogel propagated cells to better mimic *in vivo* osteosarcoma for drug treatment testing. In addition, multi-cellular tumour spheroids (MCTS) were generated from 2D primary cultures to compare 3D models created after propagation in 2D to the microsarc models. Despite the low success rate (one out of seven), we demonstrated the use and relevance of osteosarcoma 3D culture models in the context of genome informed medicine.

## Methods

### Patient samples

All tumour tissue samples used for this study were derived from the bone and soft tissue tumour biobank of the Leiden University Medical Center (BWD005/SH/sh) and obtained from patients undergoing surgical resection. Patient samples were collected between 2017 and 2019. The use of tumour tissue samples for this study was approved by the Medical Ethics Committee Leiden The Hague Delft (B16.026). Written informed consent was obtained from all participants and the study was conducted according to the code for Proper Secondary Use of Human Tissue in Netherlands. Relevant clinical information can be found in Table 1.

**Table 1:** Overview of osteosarcoma patient samples used in this study.

Patient ID	Subtype	Location	Age	Gender	Tumour sample	Neo-adjuvant treatment	Tumour specific alteration	Confirmed in 3D culture (analysis method)	Targetable alteration	Target specific drug
L6558	Conventional, telangiectatic	Fibula	36	M	Primary	Yes	EPHB1 SNV (exon 7 > chr3: 135162098 A > G); PES SNV (exon 12 > chr22: 30579877\u00B0C > T)	No (Sanger sequencing)	BRCAness signature	PARPi
L6565	Conventional	Humerus	57	F	Primary	No	KRAS SNV (c.35G > A; VAF: 0.64); CDKN2A loss	Yes (Cancer Hot Spot Panel)	CDKN2A loss	Palbociclib
L6581	Conventional	Lung	29	M	Metastasis	Yes, for treatment of primary tumour	F13A1 SNV (exon 4, >chr6: 6266605\u00B0C > T); USP43 SNV (exon 6, >chr17: 9680303 G > A)	No (Sanger sequencing)	None	NA
L6620	Conventional	Humerus	51	F	Primary	No	MTRR SNV (intron 13, >chr5: 7897041 G > T)	No (Sanger sequencing)	BRCAness signature	PARPi
L6621	Conventional, chondroblastic	Iliac bone	21	M	Primary	Yes	MYC amp	Yes (MYC IHC staining)	MYC amp	JQ1 or 10,058-F4
L6647	Conventional	Abdomen	25	M	Metastasis	No	P53 SNV (c.775G > T; VAF: 0.74)	Yes (P53 IHC staining)	4q12 amp (KIT, PDGFRA, KDR)	Regorafenib
L6727	Conventional, telangiectatic	Pelvis	67	F	Primary	Yes	P53 SNV (c.406C > T; VAF: 0.45)	No (Cancer Hot Spot Panel)	None	NA

M, male; F, female; SNV, single nucleotide variant; amp, amplification; NA, not applicable.

### Tissue processing

Tissue samples were obtained from resection specimens immediately after surgery, minced with razor blades and immersed in 3 mL 1 mg/mL collagenase IA/dispase II (C9891, Sigma-Aldrich, Saint Louis, MO, United States and #17105041 Gibco, Waltham, United States) either at 37 °C for 2 hours or at room temperature overnight. Once digested, the samples were washed twice with DMEM:F12 (#10565018, Gibco, Waltham, United States). The remaining pieces of tissue were gently broken up by pipetting with a glass pipette to obtain a cell suspension. At this stage the cells were used for 2D or 3D culture. To produce mono-layer cultures (2D) the cells were transferred into flasks and cultured in αMEM (#LO BE12-169F, Lonza, Basel, Switzerland) supplemented with 10% fetal bovine serum (FBS), 100 U/mL penicillin and 100 μg/mL streptomycin (Life Technologies Limited), and 1% NEAA (#11140050, Gibco, Waltham, United States). Cells were cultured in a humidified incubator with 1% O2 and 5% CO2 and passaged at high confluence, until a stable propagation was achieved.

### Osteosarcoma MCTS production

MCTS were produced from conventionally cultured (2D) primary osteosarcoma L6565 cells. The cells were suspended in αMEM (#LO BE12-169F, Lonza, Basel, Switzerland) supplemented with 10% fetal bovine serum (FBS) and 100 U/mL penicillin and 100 μg/mL streptomycin (Life Technologies Limited) and 1% NEAAs (#11140050, Gibco, Waltham, United States). Additional supplementation with 50 μg/mL ascorbate 2-phosphate (A8960, Sigma-Aldrich, Saint Louis, MO, United States) and 100 nM dexamethasone (D8893, Sigma-Aldrich, Saint Louis, MO, United States) was performed directly before culture. The medium contained 0.24% (w/v) methyl cellulose (M0512, Sigma-Aldrich, Saint Louis, MO, United States) to improve the spheroid reproducibility. The cells were then seeded onto pre-made 1% (w/v) agarose (#16500, Invitrogen, Waltham, United States) coated (ultra-low attachment) plates at a density of 6,000 cells/well. They were aggregated for 3 days and were subsequently used for therapeutic testing.

### Osteosarcoma 3D primary line production (hydrogels and microsarcs)

To expand the patient lines, whilst reducing risk of cell dedifferentiation during culture, we established a method for propagation of cells in a collagen-based hydrogel. After tissue digestion, the cell suspension was strained using a 70 μm cell strainer (#431751, Corning, New York, United States). If blood was present in the pellet, cells were additionally washed with PBS and treated with 4 mL RBC lysis buffer (#00-4333-57, Invitrogen, Waltham, United States) for 4 min. To stop the lysis 30 mL of PBS was added and the cells were washed in cell culture medium. The cells were suspended in a collagen scaffold (2.5 mg/mL rat tail collagen (#50202, Ibidi, Gräfelfing, Germany), 1x DMEM, 8.29 μM NaOH, 0.38% alginate (w/v)) and plated out either in 12-well (for propagation) or 96-well (for therapeutic compound testing) non-treated cell culture plates into 10 or 80 μL gels, respectively. The plates were incubated at 37 °C for 25 min and then left at room temperature for an additional 5 min before adding solution. In order to gelate the alginate component of the scaffold and further stiffen the gel, the samples were incubated in 0.2 M CaCL2 solution for 5 min. The samples were subsequently washed twice with 0.15 M NaCl solution, followed by two washes with medium and placed in culture medium containing αMEM (#LO BE12-169F, Lonza, Basel, Switzerland) supplemented with 10% FBS and 100 U/mL penicillin and 100 μg/mL streptomycin (Life Technologies Limited) and 1% NEAAs (#11140050, Gibco, Waltham, United States). Additionally, medium was supplemented with 50 μg/mL ascorbate 2-phosphate and 100 nM dexamethasone (osteogenic growth factors). Cells were grown in a humidified incubator with 1% O2 and 5% CO2 and cultured until >80% gel confluency for either passage or treatment. Cells were regularly STR-profiled using the GenePrint 10 system kit (Promega, Madison, WI, United States) and showed the same STR profile as the original tumour. In addition, all cells used for treatment were regularly tested for mycoplasma. At the end of the study, the L6565 cell line was confirmed to have no additional genetic alterations due to long-term passaging.

To passage the osteosarcoma hydrogels, cultures were first collected and washed once with PBS. In order to digest the scaffold 0.25 mg/mL LiberaseTL (#5401020001, Roche, Basel, Switzerland) solution (in PBS) was added (4,375 μL/μL scaffold). The scaffolds were incubated at 37 °C for digestion, checked and lightly vortexed after the first 10 min then subsequently at 5 min intervals until the gel was visibly dissolved and the cells were released into a single cell solution. The digestion was quenched using medium and the cells subsequently washed with medium. On a bi-passage basis the cells were strained at this stage to remove debris using autoclaved 50 μm cell strainers (#04-0042-2317, Sysmex, Kobe, Japan). The cells were then resuspended in fresh gel at a passage ratio of 1:2 for further culture or frozen for cryopreservation.

For further therapeutic testing, microsarcs were additionally produced from the L6565 hydrogel cultured cell line. The method used to produce and treat microsarcs was identical to the MCTS protocol described above, but a higher seeding density (10,000 cells/well) was used to ensure linear growth during treatment.

### Next generation sequencing data analysis

For whole exome sequencing (WES), DNA was isolated from matched pairs of primary tumour and normal frozen tissue of seven patients using the Wizard Genomic DNA purification kit (Promega, Madison, WI, United States) according to the manufacturer’s instructions. DNA samples were checked for degradation by gel electrophoresis. Whole exome sequencing with a minimum coverage of 100x was performed by GenomeScan BV (Leiden, Netherlands) using the Illumina Novaseq6000 platform and the Agilent SureSelect Human All Exon V7 kit. Raw WES data was deposited in the European Genome-phenome Archive (EGA) under the study accession number EGAS50000001583. The WES data were processed using the BioWDL somatic variant calling pipeline developed at LUMC.^1^ The quality control was first performed by FastQC (v0.11.9). Then the adapters were further clipped using Cutadapt (v2.8). The clean reads were aligned to the human reference genome GRCh38 using BWA-MEM (v0.7.17-r1188). According to the GATK best practice, duplicated mapped reads were marked using Picard (v2.20.5) and the base quality recalibration was performed using GATK4 (v 4.1.2.0) to generate the analysis-ready reads in the BAM format. Strelka (version 2.9.7) and Mutect2 (version 4.1.2.0) were used to detect small somatic variants from the matched tumour-normal samples. Only the variants meeting the criteria as determined by Strelka and Mutect2 were included in the down-stream analysis. These variants were then normalized and decomposed using vt program (v2015.11.10) and subsequently annotated using VEP (v98) with SIFT and Poly-phen-2 options enabled. VEP plugins and custom annotation files were also used to include annotations of CADD scores, dbNSFP3.5 (phastCons100way vertebrate & phy-loP100way vertebrate), population allele frequency information (e.g., gnomAD version 3 and GoNL). Copy number variants (CNVs) were identified following GATK somatic CNV discovery best practice workflow (v4.1.4.0). Pre-selected targetable genes were used to prioritize both small variants and CNV (Supplementary Table 1). Genes were labeled “targetable” when it occurred in more than one of the following targetable cancer gene panels: FoundationOne and FoundationOne Heme, MSK-IMPACT (17), Mioncoseq (18), and UCSF 500 Cancer Gene Panel.

For the customized Cancer Hotspot targeted sequencing panel, DNA was isolated from osteosarcoma hydrogels of L6727 P5 and L6565 P15 & P45 and monolayer culture of L6565 P46 using the Wizard Genomic DNA purification kit (Promega) according to the manufacturer’s instructions. Libraries were generated using Life Technology’s Ion AmpliSeq Cancer Hotspot Panel v6. Data analysis was performed as described previously. To confirm presence of non-driver tumour-specific variants in the 3D cultures, PCR and Sanger sequencing for the genes EPHB1, PES, F13A1, USP43 and MTRR was performed.

The tumour mutational burden (TMB) was calculated per sample by dividing the number of protein-coding somatic mutations by the total size of the exome (30 Mb was used which is a commonly agreed standard). Using the fit_to_signatures_strict (cutoff = 0.010) from MutationalPatterns R-package (v3.0.1), mutational signature profiles per sample were generated based on SigProfiler exome SBS reference signatures.^2^

### Immunohistochemistry

Whole slide sections of paraffin embedded tumour tissue, MCTS, hydrogels, and microsarcs (4 μm) were made. After deparaffinization and rehydration the sections were either stained using hematoxylin and eosin to observe morphology, or used for immunohistochemical staining to confirm expression of specific proteins, including SATB2 (clone CL0276, Sigma-Aldrich, Saint Louis, MO, United States), MYC (clone Y69, Abcam, Cambridge, United Kingdom) and p53 (clone DO-7, Dako, Agilent Technologies, CA, United States). Additional stainings were performed for L6565 hydrogels and MCTS: Ki67 (clone D2H10, Cell Signaling Technology), p16 (clone E6H4, CINTEC, Roche, Basel, Switzerland), and Rb (clone G3245, BD Pharmingen, San Diego, CA, United States). For SATB2 and Ki67 staining, antigen retrieval was performed by a 10 min incubation in 10 mM citrate buffer (pH 6) followed by cooling down for 2 hours. For MYC, p53, p16 and Rb staining, antigen retrieval was performed using Tris-EDTA (pH 9). Sections were incubated with primary antibody (Ki67, 1:1600; MYC, 1:80; p53, 1:1; Rb, 1:2000, SATB2, 1:10, p16, 1:1) overnight at 4 °C. The next day, sections were incubated with BrightVision one step detection system poly-HRP anti-mouse/rabbit (Immunologic, WellMed BV, Duiven, Netherlands) for 30 min. Samples were then washed with PBS and DAB+ Chromogen (Dako) was added to each slide and incubated for 10 min. Slides were counterstained with hematoxylin.

### Drug treatment

For treatment of L6565 cultures standard chemotherapeutic agents used in sarcomas, doxorubicin and cisplatin were included, while palbociclib, a CDK4/CDK6 inhibitor, was used to target the homozygous CDKN2A loss.

For therapeutic treatment of monolayer cell cultures, L6565 cells were seeded at 4,000 cells per well of a 96-well plate. For monotherapy treatment, cells were treated the following day for 72 h with palbociclib (Selleckchemicals, Houston, TX, United States), doxorubicin or cisplatin (both in-house hospital pharmacy of Leiden University Medical Center), in concentrations ranging from 0.00001 to 100 μM. For combination treatments, cells were seeded in medium containing cisplatin or doxorubicin for 24 h. The following day, cisplatin or doxorubicin was removed, and palbociclib was added for 72 h. Pretreatment of cisplatin and doxorubicin was based on a previously published study indicating that simultaneous addition of chemotherapies with palbociclib will result in interference between the drugs (19).

L6565 MCTS and microsarc cultures were treated with palbociclib for 72 h (3 days) or 7 days in concentrations ranging from 0.001 to 100 μM. For the comparison of long-term palbociclib treatment (7 days in total) the drug medium was refreshed after 3 days of treatment.

After drug treatment, cell viability was determined by Presto Blue Cell Viability reagent after a 90 min (for MCTS and microsarcs) or 60 min (for monolayer cultured cells) incubation, after which fluorescence was measured at 550/600 nm using a microplate reader (Infinite M Plex, Tecan Group Ltd., Zürich, Switzerland). Osteosarcoma hydrogels were formalin fixed and paraffin embedded after read-out. The readout was used to determine the drug concentration that inhibited cell viability by 50% (IC50) and to determine synergy of the combination therapies. The Bliss independence model (C = A + B – A × B) was used to predict synergy, in which C represents the combined effect and A and B represent single agent effects (20). A heatmap figure was created with MORPHEUS (Broad Institute, Cambridge, MA, United States).

## Results

### Detection of targetable alterations in the osteosarcoma patient cohort

**Figure 1:**
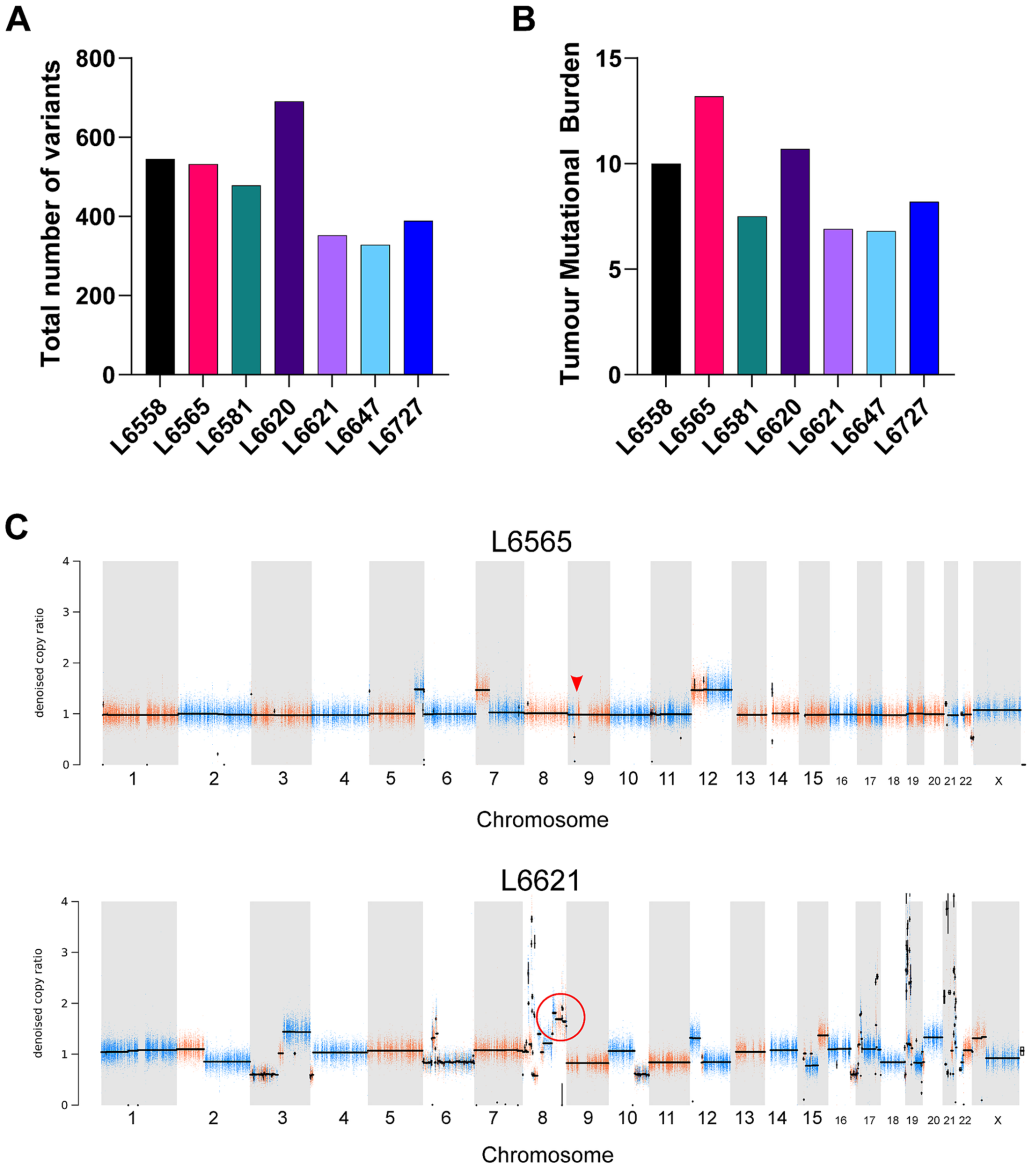
Whole exome sequencing analysis of osteosarcoma tumour samples. **(A)** Total number of somatic single nucleotide variants per tumour. Bars represent the number of overlapping variants identified by different variant callers (Strelka, Mutect2). **(B)** Tumour mutational burden per patient. Values are expressed as mutations per megabase. **(C)** Copy number profiles of L6565 and L6621. For L6565 the copy number loss of *CDKN2A* is indicated with a red arrow. For L6621 the *MYC* amplification is indicated with a red circle.

In total, seven patients were included (Table 1). Tissue specimens were harvested from primary or metastatic sites. Three patients with a primary tumour received neo-adjuvant chemotherapy. Various histological variants, including telangiectatic and chondroblastic osteosarcoma, were represented (Table 1). Whole Exome Sequencing (WES) revealed that the total number of single nucleotide variants of each tumour varied among patients (Figure 1A). The tumour mutational burden in all osteosarcoma patients was low to intermediate (average 9.0 mutations per megabase) (Figure 1B) (21). The copy number profiles confirmed the presence of highly complex genomes with many copy number alterations, although the level of complexity varied, with the exception of L6565 that only showed a small number of copy number changes (Figure 1C; Supplementary Figure 1). Mutational signature analysis revealed that two COSMIC mutational signatures were dominant (signature SBS5 and SBS31, Supplementary Figure 2), although these were not associated with a specific etiology. In L6558 and L6620 the mutational signature SBS3 was identified, albeit at a low level, which is associated with a BRCAness signature, and may suggest sensitivity to PARP inhibitors (22). Both tumours did not show alterations in homologous recombination deficiency related genes (23).

All seven osteosarcomas were analyzed for the presence of targetable alterations (Table 1) by filtering genetic alterations including single nucleotide variants and copy number alterations using a predefined list of targetable genes (Supplementary Table 1). Potential molecular targets were identified for five out of seven patients (Supplementary Table S2). L6565 showed a KRAS mutation (c.35G > A; VAF: 0.64) and homozygous loss of CDKN2A. The latter alteration is targetable by CDK4/CDK6 inhibitors such as palbociclib. L6621 harbored a MYC amplification, which can be targeted by MYC inhibitors such as JQ1 and 10058-F4. L6647 carried an amplification in the region 4q12, which contains the genes KIT, PDGFRA and KDR which may be targeted by receptor tyrosine kinase inhibitors such as regorafenib. For two tumours, L6581 and L6727, no targetable alterations were identified. Thus, the cohort of seven patients reflected the molecular heterogeneity that is characteristic of osteosarcoma.

### Long-term genetically stable 3D osteosarcoma hydrogel cultures can be established from human osteosarcoma resection material

**Figure 2:**
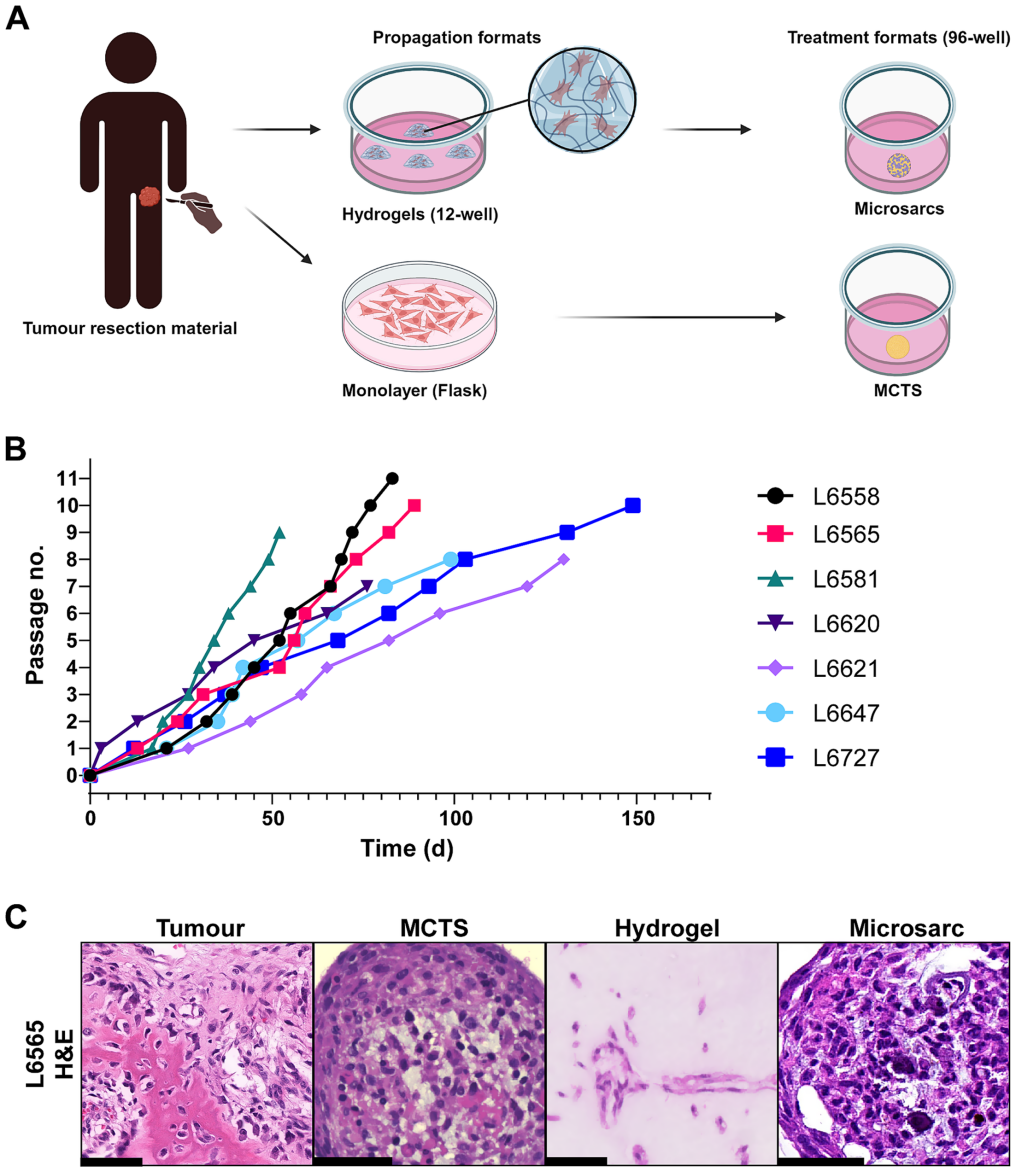
Growth rate and histology of 3D osteosarcoma cultures. **(A)** Osteosarcoma patient resection material was digested to single-cell suspensions, followed by propagation in a collagen-based scaffold with optimized osteosarcoma medium (hydrogels) or cultured in monolayer (2D). For treatment of cells propagated in hydrogels, cells were subsequently seeded as microsarcs. For treatment of cultures derived from monolayer, cells were seeded as multicellular tumour spheroids (MCTS). **(B)** Seven osteosarcoma lines were established and cultured for at least seven passages without signs of a growth plateau. **(C)** H&E staining of L6565 original tumour and corresponding MCTS, hydrogel, and microsarc cultures. Scale bar represents 50 μm.

Monolayer and hydrogel culture was performed for patient material propagation, followed by assessment of monolayer, MCTS, hydrogel and microsarc cultures as treatment formats (Figure 2A). For all seven osteosarcomas hydrogel lines were cultured in collagen-based scaffolds over the course of 44–120 days to at least passage 7 (Figure 2B). As all cultures were passaged at a ratio of 1:2 once confluent, we approximated the doubling time based on passaging frequency, which ranged from 6 to 26 days, dependent on the patient line. Monolayer culture could only be established for L6565. During the COVID-19 pandemic, all experimental work was suspended and all of the patient line cultures were cryopreserved, which affected the recovery and propagation time of all of the patient lines, except for L6565 which could be continuously passaged at the same rate.

L6565 was then used to compare monolayer, MCTS, hydrogel and microsarc culture to determine the most representative model for therapeutic testing. Hydrogel cultures showed the lowest cell density as compared to MCTS or microsarcs. Morphology of the cells in culture was reminiscent of the original tumour, showing spindle-shaped morphology (Figure 2C). However, none of the L6565 patient-derived lines displayed clear evidence of osteoid deposition, and SATB2 immunohistochemistry, a marker for osteogenic differentiation, was negative in MCTS, hydrogel, and microsarc cultures (Supplementary Figure 3). Of note, higher passages (i.e., p45) of L6565 hydrogel cultures retained the observed KRAS variant (VAF = 0.66) and homozygous CDKN2A loss without acquiring additional mutations, indicating a stable culture was established that shows minimal genetic drift whilst culturing for a longer period of time.

### Molecular confirmation of the presence of tumour cells in the 3D cultures

To confirm the presence of tumour cells in later passages (>p5), or to exclude overgrowth of other cell types present in the tumour tissue, the presence of tumour specific alterations identified in the primary tumour was investigated in the hydrogel cultures. Since specific recurrent aberrations are lacking in osteosarcoma, this had to be customized per patient. Using Cancer Hotspot targeted next generation sequencing, we confirmed that L6565 osteosarcoma hydrogels contained the identical KRAS mutation and homozygous loss of CDKN2A as the original tumour (Supplementary Figure 4). Moreover, L6565 osteosarcoma hydrogels showed loss of p16 protein expression (Supplementary Figure 5). Because no driver mutations were available for patient lines L6727, L6558, L6620, and L6581, other somatic variants were used to compare the primary tumour with the 3D cultures. Single nucleotide variants were selected on the basis of coverage (>100) and variant allele frequency (>0.3) and include a TP53 point mutation (c.406C > T, VAF: 0.45), a EPHB1 point mutation, a MTRR point mutation, and a F13A1 point mutation, respectively (Table 1). These mutations were not found in the 3D cultured cells, suggesting that tumour cells were overgrown by other cell populations within the cultures or, less likely, that the mutations were lost via selection, meaning these cultures cannot be considered as validated osteosarcoma models. For L6621 and L6647 respectively, the positive immunohistochemical staining for MYC and p53 suggested the presence of tumour cells, though due to lack of sufficient cells after cryostorage we were unable to confirm this on a genetic level (Supplementary Figure 5). Thus, one out of seven 3D osteosarcoma hydrogel cultures contained detectable, target representative tumour cells that remained viable after cryostorage and could be long term expanded, leading to a 14% final success rate (Table 1). Therefore, only L6565 cultures could be used to experimentally test its identified genome-informed therapy.

### L6565 is sensitive to palbociclib

**Figure 3:**
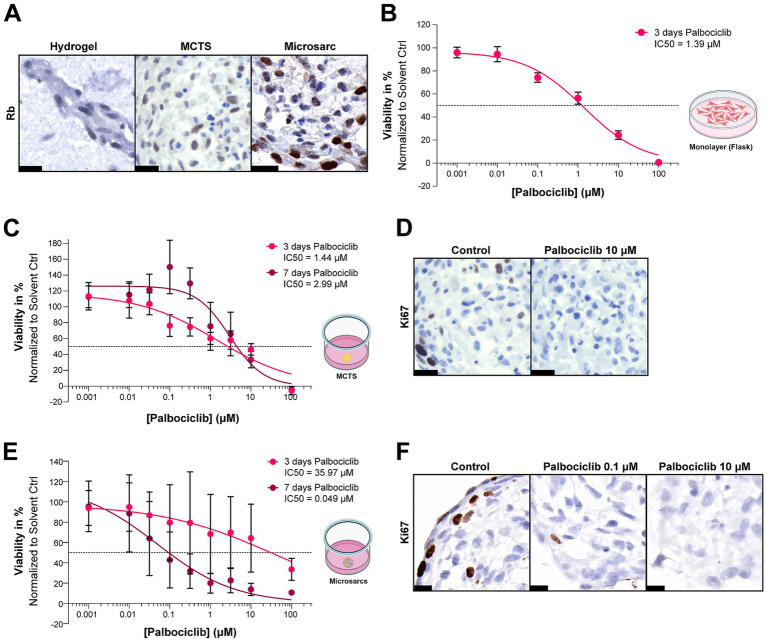
Palbociclib treatment of L6565 cultures. **(A)** L6565 MCTS, hydrogels, and microsarcs stained positive for Rb. Scalebar represents 20 μm. **(B)** Cells cultured in monolayer were treated with palbociclib for 72 h, after which cell viability and IC50 values were determined. The graph represents the average of two experiments performed in triplicate, with the standard deviation. **(C)** L6565 MCTS were treated with palbociclib for 3 days (72 h) or 7 days, after which cell viability and IC50 values were determined. The graph represents the average of three experiments performed in triplicate, with the standard deviation. **(D)** Immunohistochemistry shows a decrease in Ki67 positive cells when treated with 10 μM of palbociclib compared to untreated control. Scalebar represents 20 μm. **(E)** L6565 microsarcs were treated with palbociclib for 3 or 7 days, after which cell viability was determined. Bars represent the average of four experiments performed in triplicate, with the standard deviation. **(F)** Immunohistochemistry shows a decrease in Ki67 positive cells when treated with 0.1 or 10 μM of palbociclib for 7 days compared to untreated control. Scalebar represents 10 μm.

The CDKN2A deletion in L6565 suggested potential vulnerability to CDK4 and −6 inhibition using palbociclib. For palbociclib to be effective, Rb should be functional so its presence was confirmed in L6565 hydrogel, MCTS and microsarc cultures by immunohistochemistry (Figure 3A). Palbociclib treatment of L6565 monolayer cultured cells induced a dose dependent decrease in viability after 72 h of treatment (IC50 = 1.39 μM) (Figure 3B). To reduce the effective dose of palbociclib, we investigated combination therapy with two common chemotherapies (doxorubicin and cisplatin) which also had a dose dependent effect on the viability of monolayer cultured cells (IC50 = 0.11 and 2.30 μM respectively), however no pronounced synergy was identified using Excess over Bliss calculations, therefore we did not explore combination therapy further (Supplementary Figure 6). Palbociclib treatment was then tested on MCTS cultures of L6565, which were generated and treated for the standard 72 h (3 days) as well as 7 days. The cells showed decreased viability in response to palbociclib, though there was no evident difference in the IC50 values between 3 and 7 days (IC50 = 1.44 μM vs. 2.99 μM) (Figure 3C). Morphological evaluation showed the presence of viable tumour cells, though Ki67 immunohistochemistry confirmed a decrease in proliferating cells (Figure 3D). However, the same treatment regime for microsarc culture yielded differing IC50 values (IC50 = 35.97 μM vs. 0.049 μM) (Figure 3E), again with a reduction of proliferating cells at lower treatment concentration after 7 days treatment (Figure 3F). The L6565 microsarcs were arguably more sensitive to 7 days drug treatment compared to L6565 cells cultured in monolayer or as MCTS (Figure 3E) although the experimental variability had also increased.

## Discussion

Further improvement of osteosarcoma patient survival is severely hampered by the high interpatient molecular heterogeneity. Over the past decades, multiple studies have defined distinct osteosarcoma molecular subgroups (24–27). *In vitro* or *in vivo* models representing these various molecular subtypes are currently lacking but urgently needed. While cell lines and 2D, confluent cultures are often used, 3D culture models more closely mimic the *in vivo* situation. For this purpose, we explored for seven osteosarcoma patients the feasibility of establishing long term osteosarcoma 3D cultures straight from osteosarcoma patient resection material to test genome informed therapies.

The genomic findings in the seven osteosarcoma patients reflected the known molecular heterogeneity of osteosarcoma. Potential therapeutic targets were identified in five tumours. From all seven patients osteosarcoma hydrogels were established and propagated until at least seven passages. Osteosarcoma hydrogel production methods used in the current study are tailored to mimic the osteosarcoma microenvironment (collagen hydrogels and osteogenic growth factors), single-cell seeded and utilize less additional supplements compared to culture methods frequently used for organoid culture of epithelial cancers. Supplementation in a sarcoma context was expected to lead to a more representative culture of osteosarcoma patient samples. Additionally, although in line with other organoid protocols, the level of antibiotics used for 3D culture should be as low as possible in future experiments, as they may have an effect on response to therapy (28).

In four of seven cultures molecular alterations were lacking, indicating that the cells were either overgrown by other cell types, or, less likely, that key alterations were lost at higher passages, and therefore these established cultures cannot be considered as validated osteosarcoma models. Fibroblast overgrowth is a well-known problem in cell culture for mesenchymal tumours, and it can be difficult to distinguish these cells from tumour cells. Our study highlights the importance of confirming the presence of tumour cells at the genetic level when using cell culture. This can be particularly challenging, requiring a personalized approach, in case no general tumour markers are available, as is the case in osteosarcoma. L6565 cultures harbored the same genetic alterations as the original tumour, and no additional genetic alterations were found over the course of the culture use, confirming stable presence of tumour cells. Two other patient lines, L6621 and L6647, showed indication that targetable genetic alteration existed through IHC, however we did not have enough material after cryostorage to confirm this at the genetic level. Although some cultures harbored tumour cells, osteosarcoma characteristics such as osteoid deposition and SATB2 positivity (a marker for osteogenic differentiation) were lacking in all culture formats, suggesting additional optimization of culture conditions is needed to more closely mimic tumours in patients. Successful culture of tumour cells instead of fibroblasts was unrelated to tumour status (primary/recurrence/metastasis) or treatment and as such there were no specific characteristics that could predict success. L6565 was most successful, both in monolayer as well as in the 3D models, although this osteosarcoma is slightly unusual with regards to clinical presentation and absence of the typical complex genome. The patient concerned a 57 year old female with a pathological fracture of the left humerus and a lesion in the left clavicle. Biopsies of the clavicle as well as the humerus revealed similar morphology displaying a low-grade undifferentiated spindle cell sarcoma without deposition of osteoid, and MDM2 amplification as determined by FISH was lacking. First, the 6 cm lesion in the clavicle was resected, showing a heterogeneous morphology, including an intermediate grade area with deposition of osteoid, resulting in the diagnosis of osteosarcoma. Resection of the humerus was performed, revealing a 20 cm tumour with similar morphology but also including areas of conventional high-grade osteosarcoma in addition to high grade spindle cell areas without deposition of osteoid. No fusions were detected using Archer fusionplex sarcoma analysis in routine diagnostics.

Unfortunately, after the COVID-19 pandemic, only L6565 was able to recover from the freeze–thaw process contributing to the low success rate of 1/7 (14%), suggesting that osteosarcoma tissue may be particularly sensitive to cryopreservation. Future studies should address the improvement of cryopreservation methods to enable longer term maintenance. The difference between standard and specialized cryopreservation approaches was investigated in the past in the context of organoid culture and xenografts, finding a marked benefit with use of specialized freezing medium such as Cryostor^®^ (29).

Long-term (<6 months) growth of osteosarcoma lung metastases was demonstrated previously by He and colleagues, though molecular analysis was lacking (14). Our feasibility study did not address short term culture, which may provide an alternative when used for genome informed drug testing. However, growth rates are slow, and it will be difficult to collect enough cells to confirm the presence of tumour cells among fibroblasts.

L6565 could be cultured long term and carried a homozygous loss of CDKN2A, with intact Rb, thereby suggesting vulnerability to CDK4/CDK6 inhibitors. L6565 cultures were indeed sensitive to the CDK4/CDK6 inhibitor palbociclib, providing a proof-of-concept for using patient derived 3D osteosarcoma cultures to test genome-informed therapies. The results obtained in this singular patient-derived model are in agreement with previous clinical studies showing that a subset of osteosarcomas are vulnerable to palbociclib (4, 30–32). *In vitro* doses used by us and others to achieve an effect are however relatively high (0.5–27 μM concentration range) and exceed plasma levels observed in patients treated with palbociclib, suggesting limited potential for clinical translation (30, 31, 33). However, increasing treatment duration to 7 days reduced the effective concentration of palbociclib in L6565 microsarc cultures within the range of clinically observed Cmax plasma levels (0.101 μM) (34), which are reached by a daily 125 mg palbociclib oral dose regimen typically used in clinical trials involving palbociclib (NCT03242382, NCT03526250). Nevertheless, it seems that current palbociclib dosing regimens are not sufficient to elicit a clinical response (35, 36). Combination with other therapeutic agents was suggested, however in this study combination therapy with doxorubicin or cisplatin did not significantly increase sensitivity to palbociclib and therefore the dose could not be lowered with this approach. There are currently two clinical trials ongoing that test CDK4/CDK6 inhibitors in osteosarcoma patients with a known alteration in the CDK4/CDK6 pathway (NCT03242382, NCT04040205), one of which is employing an alternative inhibitor, abemaciclib, which may show higher efficacy. Abemaciclib and other FDA approved CDK4/CDK6 inhibitors (ribociclib and trilaciclib) should be further tested in the L6565 microsarc model to determine if these alternative treatments show improved pharmacokinetic/pharmacodynamic features to enhance potential for clinical translation. Moreover, the CDK4/CDK6 inhibitors should be tested in additional patient derived 3D osteosarcoma cultures to explore if CDKN2A loss with intact Rb could serve as a predictive biomarker for response in osteosarcoma patients.

Drug responses were compared between osteosarcoma monolayer, MCTS, and microsarc cultures. Sensitivity to palbociclib was demonstrated in all of the models, however the more complex microsarc model, made from cells where dedifferentiation was reduced by propagation of the cells in an extracellular matrix matched to the tumour microenvironment, was significantly more variable in response to the treatment. Since we compared this response against MCTS which are 2D propagated 3D cultures, we can assume with higher confidence that this variability could be due to biological variation rather than technical, as the seeding and treatment parameters for the two are the same and only the handling of the cells before this stage differs. Of course, some variability could be attributed to the difference in passaging protocol, however we tested the effect of the harshest step (Liberase TL treatment duration) which showed an insignificant level of variability (data not shown). This suggests that the increased variability observed in microsarc cultures may be biological, due to better preservation of culture heterogeneity. The current study did not assess the presence of different cell populations within the established cultures and additional, single cell approaches would be needed to determine if the established cultures reflect the cellular composition of the original tumour. The minor difference in sensitivity observed between monolayer and MCTS cultured cells can be explained by the increase in tight intercellular junctions which occur in 3D cultures and reduce drug penetration, as well as the higher presence of extracellular matrix components (37). However, the difference in sensitivity in microsarc cultures compared to monolayer propagated cells transitioned to a 3D environment (MCTS) might be explained by additional changes occurring upon continuous culturing on a 2D plastic surface (38, 39). Further investigation into the benefits of 3D primary culture models that have never been in contact with a plastic surface, is required to determine whether there is an increased representativity over 3D models propagated using cells first cultivated in monolayer.

In conclusion, the current feasibility study shows that the generation of 3D cultures straight from osteosarcoma tissue is feasible, but highly challenging. It is labor intensive, the success rate is low, and the growth rate is slow, similar to what has been recently reported for other sarcoma subtypes (13). Moreover, due to the lack of a tumour specific marker for osteosarcoma, confirmation of the presence of tumour cells, which is imperative to exclude overgrowth of fibroblasts, needs to be personalized. While we focussed on the establishment of osteosarcoma long term 3D cultures, for use in clinical practice the turnaround time should be decreased and culture conditions would need optimization to allow short-term assessment of each patient-derived culture without 2D propagation. Freeze–thaw cycles should be avoided or further optimized, as osteosarcoma cells are quite vulnerable. Nevertheless, this study provides a proof-of-concept for evaluating genome-informed therapies in patient derived 3D osteosarcoma models. 3D primary cultures that have never been in contact with a 2D plastic surface, such as the ones used in this study, may be more representative of osteosarcoma *in vivo* and should be studied further in order to determine optimal *in vitro* models for drug discovery pipeline integration.

## Data Availability

The datasets presented in this study can be found in online repositories. The names of the repository/repositories and accession number(s) can be found in the article/Supplementary material. Raw WES data was deposited in the European Genome-phenome Archive (EGA) under the study accession number EGAS50000001583.

## Footnotes

1
https://github.com/biowdl/germline-DNA/blob/v3.0.0/somatic.wdl

2
https://www.synapse.org/#!Synapse:syn12026190

## References

[1] Baumhoer D Böhling T Cates J Cleton-Jansen A Hogendoorn P O'Donnell P . "Osteosarcoma". In: Nielsen G, editor. Pathology and Genetics of Tumours of Soft Tissue and Bone. Who Classification of Tumours of Soft Tissue and Bone, 5th Edn. Lyon, France: IARC Press (2020)

[2] Smrke A Anderson PM Gulia A Gennatas S Huang PH Jones RL. Future directions in the treatment of osteosarcoma. Cells. (2021) 10:172. doi: 10.3390/cells10010172, 33467756 PMC7829872

[3] Gounder MM Agaram NP Trabucco SE Robinson V Ferraro RA Millis SZ . Clinical genomic profiling in the Management of Patients with soft tissue and bone sarcoma. Nat Commun. (2022) 13:3406. doi: 10.1038/s41467-022-30496-0, 35705558 PMC9200814

[4] Sayles LC Breese MR Koehne AL Leung SG Lee AG Liu H-Y . Genome-informed targeted therapy for osteosarcoma. Cancer Discov. (2019) 9:46–63. doi: 10.1158/2159-8290.Cd-17-1152, 30266815 PMC7134333

[5] Frankenbach-Désor T Niesner I Ahmed P Dürr HR Klein A Knösel T . Tissue-engineered patient-derived osteosarcoma models dissecting tumour-bone interactions. Cancer Metastasis Rev. (2024) 44:8. doi: 10.1007/s10555-024-10218-2, 39592467 PMC11599440

[6] Bassi G Rossi A Campodoni E Sandri M Sarogni P Fulle S . 3d tumor-engineered model replicating the osteosarcoma stem cell niche and in vivo tumor complexity. ACS Appl Mater Interfaces. (2024) 16:55011–26. doi: 10.1021/acsami.4c02567, 39353598 PMC11492322

[7] González Díaz EC Lee AG Sayles LC Feria C Sweet-Cordero EA Yang F. A 3d osteosarcoma model with bone-mimicking cues reveals a critical role of bone mineral and informs drug discovery. Adv Healthc Mater. (2022) 11:e2200768. doi: 10.1002/adhm.202200768, 35767377 PMC10162498

[8] Han X Cai C Deng W Shi Y Li L Wang C . Landscape of human organoids: ideal model in clinics and research. Int J Hydrogen Energ. (2024) 5:100620. doi: 10.1016/j.xinn.2024.100620, 38706954 PMC11066475

[9] Brancato V Oliveira JM Correlo VM Reis RL Kundu SC. Could 3d models of cancer enhance drug screening? Biomaterials. (2020) 232:119744. doi: 10.1016/j.biomaterials.2019.119744 31918229

[10] Abuwatfa WH Pitt WG Husseini GA. Scaffold-based 3d cell culture models in Cancer research. J Biomed Sci. (2024) 31:7. doi: 10.1186/s12929-024-00994-y, 38221607 PMC10789053

[11] Abbas ZN Al-Saffar AZ Jasim SM Sulaiman GM. Comparative analysis between 2d and 3d colorectal Cancer culture models for insights into cellular morphological and transcriptomic variations. Sci Rep. (2023) 13:18380. doi: 10.1038/s41598-023-45144-w, 37884554 PMC10603139

[12] Mohseny AB Machado I Cai Y Schaefer K-L Serra M Hogendoorn PCW . Functional characterization of osteosarcoma cell lines provides representative models to study the human disease. Lab Investig. (2011) 91:1195–205. doi: 10.1038/labinvest.2011.72, 21519327

[13] De Cock L Palubeckaite I Bersani F Faehling T Pasquali S Umbaugh S . Establishment of patient-derived 3d in vitro models of sarcomas: literature review and guidelines on behalf of the fortress working group. Neoplasia. (2025) 65:101171. doi: 10.1016/j.neo.2025.101171, 40324303 PMC12104653

[14] He A Huang Y Cheng W Zhang D He W Bai Y . Organoid culture system for patient-derived lung metastatic osteosarcoma. Med Oncol. (2020) 37:105. doi: 10.1007/s12032-020-01429-y, 33079257

[15] Nie J-H Yang T Li H Li S Li T-T Ye H-S . Frequently expressed Glypican-3 as a promising novel therapeutic target for osteosarcomas. Cancer Sci. (2022) 113:3618–32. doi: 10.1111/cas.15521, 35946078 PMC9530858

[16] Al Shihabi A Tebon PJ Nguyen HTL Chantharasamee J Sartini S Davarifar A . The landscape of drug sensitivity and resistance in sarcoma. Cell Stem Cell. (2024) 31:1524–42.e4. doi: 10.1016/j.stem.2024.08.010, 39305899 PMC12318355

[17] Cheng DT Mitchell TN Zehir A Shah RH Benayed R Syed A . Memorial Sloan Kettering-integrated mutation profiling of actionable Cancer targets (Msk-Impact): a hybridization capture-based next-generation sequencing clinical assay for solid tumor molecular oncology. J Mol Diagn. (2015) 17:251–64. doi: 10.1016/j.jmoldx.2014.12.006, 25801821 PMC5808190

[18] Mody RJ Wu Y-M Lonigro RJ Cao X Roychowdhury S Vats P . Integrative clinical sequencing in the Management of Refractory or relapsed Cancer in youth. JAMA. (2015) 314:913–25. doi: 10.1001/jama.2015.10080, 26325560 PMC4758114

[19] Salvador-Barbero B Álvarez-Fernández M Zapatero-Solana E El Bakkali A Menéndez MC López-Casas PP . Cdk4/6 inhibitors impair recovery from cytotoxic chemotherapy in pancreatic adenocarcinoma. Cancer Cell. (2020) 37:340–53.e6. doi: 10.1016/j.ccell.2020.01.007, 32109375

[20] Borisy AA Elliott PJ Hurst NW Lee MS Lehár J Price ER . Systematic discovery of multicomponent therapeutics. Proc Natl Acad Sci USA. (2003) 100:7977–82. doi: 10.1073/pnas.1337088100, 12799470 PMC164698

[21] Chalmers ZR Connelly CF Fabrizio D Gay L Ali SM Ennis R . Analysis of 100,000 human cancer genomes reveals the landscape of tumor mutational burden. Genome Med. (2017) 9:34. doi: 10.1186/s13073-017-0424-2, 28420421 PMC5395719

[22] Póti Á Gyergyák H Németh E Rusz O Tóth S Kovácsházi C . Correlation of homologous recombination deficiency induced mutational signatures with sensitivity to Parp inhibitors and cytotoxic agents. Genome Biol. (2019) 20:240. doi: 10.1186/s13059-019-1867-0, 31727117 PMC6857305

[23] Cohen D Hondelink LM Solleveld-Westerink N Uljee SM Ruano D Cleton-Jansen A-M . Optimizing mutation and fusion detection in Nsclc by sequential DNA and Rna sequencing. J Thorac Oncol. (2020) 15:1000–14. doi: 10.1016/j.jtho.2020.01.019, 32014610

[24] Jiang Y Wang J Sun M Zuo D Wang H Shen J . Multi-omics analysis identifies osteosarcoma subtypes with distinct prognosis indicating stratified treatment. Nat Commun. (2022) 13:7207. doi: 10.1038/s41467-022-34689-5, 36418292 PMC9684515

[25] Zhang H Wang T Gong H Jiang R Zhou W Sun H . A novel molecular classification method for osteosarcoma based on tumor cell differentiation trajectories. Bone Res. (2023) 11:1. doi: 10.1038/s41413-022-00233-w, 36588108 PMC9806110

[26] Shu Y Peng J Feng Z Hu K Li T Zhu P . Osteosarcoma subtypes based on platelet-related genes and tumor microenvironment characteristics. Front Oncol. (2022) 12:941724. doi: 10.3389/fonc.2022.941724, 36212395 PMC9539847

[27] Lv Y Wu L Jian H Zhang C Lou Y Kang Y . Identification and characterization of aging/senescence-induced genes in osteosarcoma and predicting clinical prognosis. Front Immunol. (2022) 13:13. doi: 10.3389/fimmu.2022.997765, 36275664 PMC9579318

[28] Ryu AH Eckalbar WL Kreimer A Yosef N Ahituv N. Use antibiotics in cell culture with caution: genome-wide identification of antibiotic-induced changes in gene expression and regulation. Sci Rep. (2017) 7:7533. doi: 10.1038/s41598-017-07757-w, 28790348 PMC5548911

[29] Ivanics T Bergquist JR Liu G Kim MP Kang Y Katz MH . Patient-derived xenograft cryopreservation and reanimation outcomes are dependent on cryoprotectant type. Lab Investig. (2018) 98:947–56. doi: 10.1038/s41374-018-0042-7, 29520054 PMC6072591

[30] Perez M Muñoz-Galván S Jiménez-García MP Marín JJ Carnero A. Efficacy of Cdk4 inhibition against sarcomas depends on their levels of Cdk4 and P16ink4 mrna. Oncotarget. (2015) 6:40557-74. doi: 10.18632/oncotarget.5829, 26528855 PMC4747352

[31] Franceschini N Gaeta R Krimpenfort P de Briaire- Bruijn I Kruisselbrink AB Szuhai K . A murine mesenchymal stem cell model for initiating events in osteosarcomagenesis points to Cdk4/Cdk6 inhibition as a therapeutic target. Lab Investig. (2021) 102:391–400. doi: 10.1038/s41374-021-00709-z 34921235 PMC8964417

[32] Delmore JE Issa GC Lemieux ME Rahl PB Shi J Jacobs HM . Bet bromodomain inhibition as a therapeutic strategy to target C-Myc. Cell. (2011) 146:904–17. doi: 10.1016/j.cell.2011.08.017, 21889194 PMC3187920

[33] Zhou Y Shen JK Yu Z Hornicek FJ Kan Q Duan Z. Expression and therapeutic implications of cyclin-dependent kinase 4 (Cdk4) in osteosarcoma. Biochim Biophys Acta (BBA) - Mol Basis Dis. (2018) 1864:1573–82. doi: 10.1016/j.bbadis.2018.02.004, 29452249

[34] Liston DR Davis M. Clinically relevant concentrations of anticancer drugs: a guide for nonclinical studies. Clin Cancer Res. (2017) 23:3489–98. doi: 10.1158/1078-0432.CCR-16-3083, 28364015 PMC5511563

[35] Macy ME Mody R Reid JM Piao J Saguilig L Alonzo TA . Palbociclib in solid tumor patients with genomic alterations in the Cyclind-Cdk4/6-Ink4a-Rb pathway: Results from National Cancer Institute-children's oncology group pediatric molecular analysis for therapy choice trial arm I (Apec1621i). JCO Precis Oncol. (2024) 8:e2400418. doi: 10.1200/PO-24-00418, 39298716 PMC11488755

[36] Zeverijn LJ Looze EJ Thavaneswaran S van Berge Henegouwen JM Simes RJ Hoes LR . Limited clinical activity of palbociclib and ribociclib monotherapy in advanced cancers with cyclin D-Cdk4/6 pathway alterations in the Dutch Drup and Australian Most trials. Int J Cancer. (2023) 153:1413–22. doi: 10.1002/ijc.34649, 37424386

[37] Gong X Lin C Cheng J Su J Zhao H Liu T . Generation of multicellular tumor spheroids with microwell-based agarose scaffolds for drug testing. PLoS One. (2015) 10:e0130348. doi: 10.1371/journal.pone.0130348, 26090664 PMC4474551

[38] Tibbitt MW Anseth KS. Hydrogels as extracellular matrix mimics for 3d cell culture. Biotechnol Bioeng. (2009) 103:655–63. doi: 10.1002/bit.22361, 19472329 PMC2997742

[39] Von Der Mark K Gauss V Von Der Mark H MÜLler P. Relationship between cell shape and type of collagen synthesised as chondrocytes lose their cartilage phenotype in culture. Nature. (1977) 267:531–2. doi: 10.1038/267531a0, 559947

