# Supplementary material for: Osteosarcoma 3D patient derived cultures to test genome-informed personalized treatment options: a feasibility study
Source: Front Med (Lausanne). 2026 Mar 17;13:1754270. doi: 10.3389/fmed.2026.1754270 (PMC13036131; doi:10.3389/fmed.2026.1754270)
Supplement: Supplementary file 1 [file Data_Sheet_1.PDF]

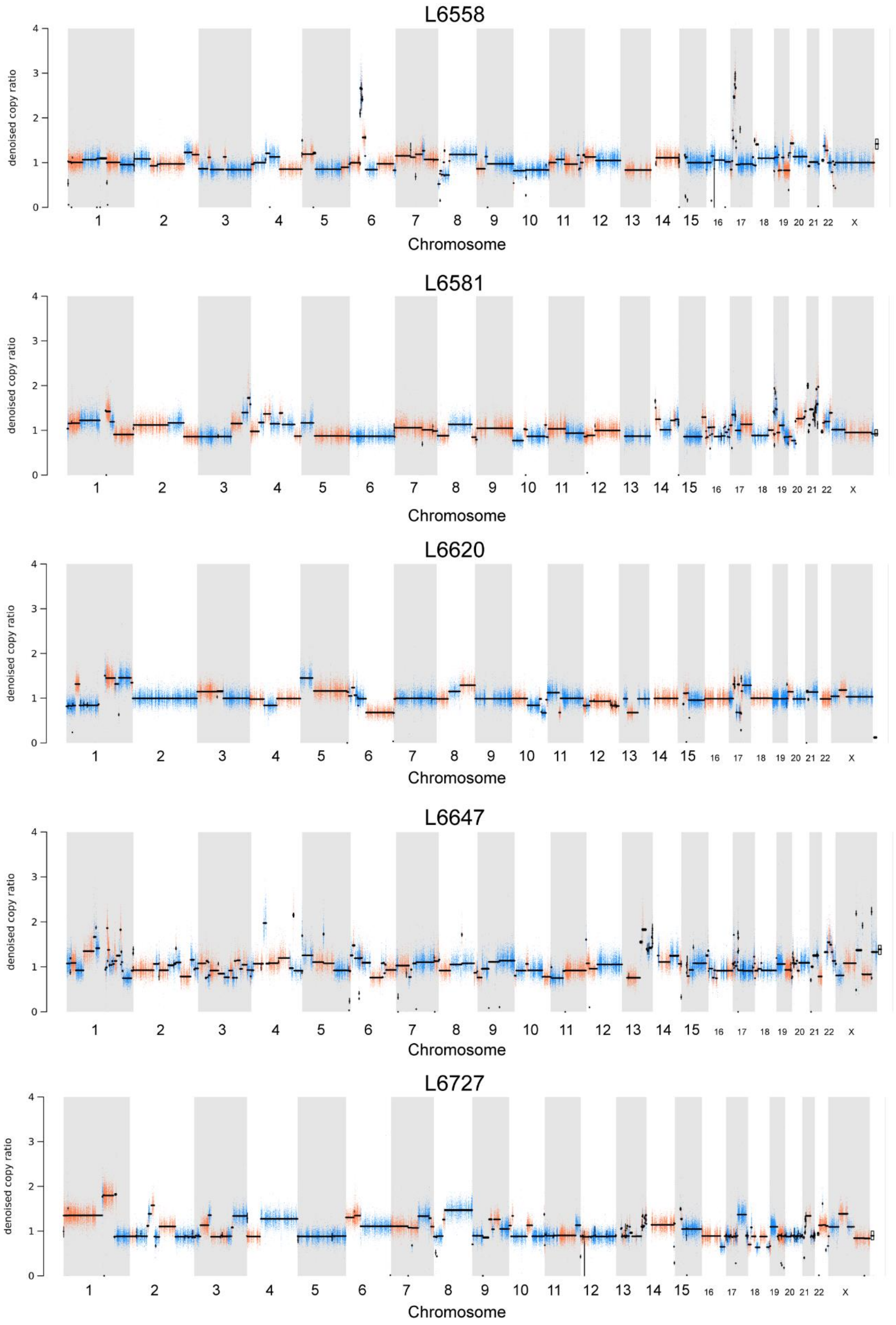

Supplementary Figure 1. Copy number profiles of L6558, L6581, L6620, L6647 and L6727.

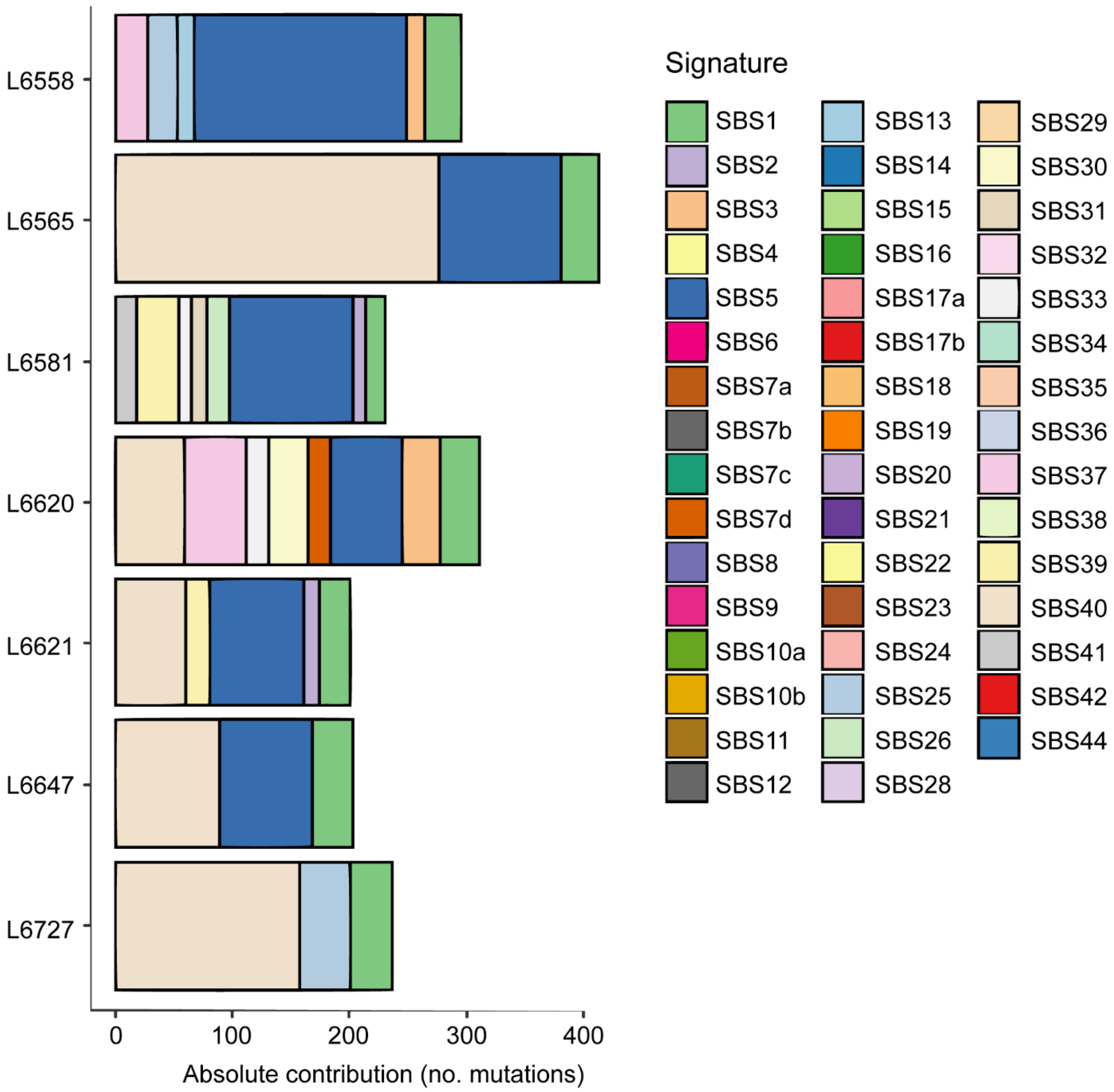

Supplementary Figure 2. Mutational signatures for all osteosarcoma samples, based on SBS reference signatures.

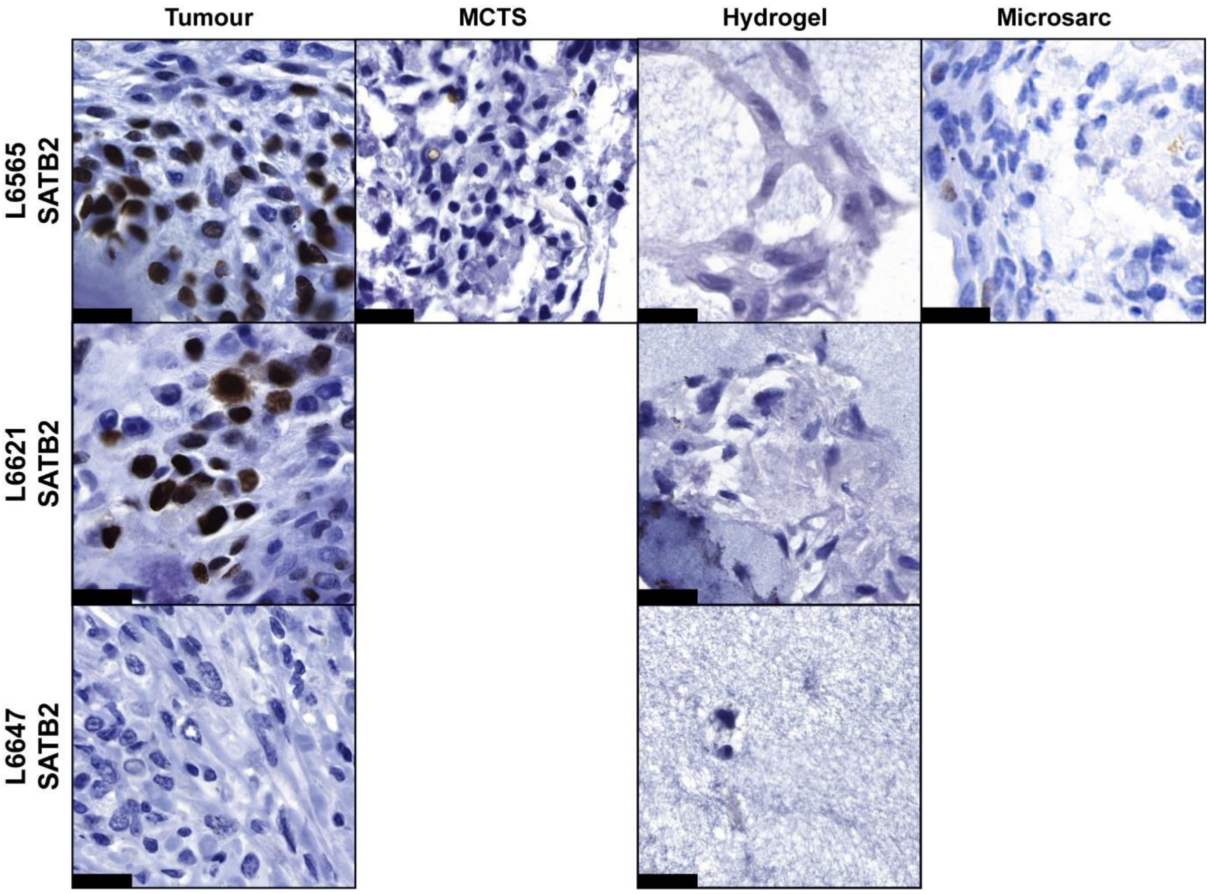

Supplementary Figure 3. SATB2 staining of osteosarcomas L6565, L6621 and L6647 and the corresponding 3D cultures (MCTS, hydrogel, microsarcs). Only these patient samples were stained for SATB2. Scalebar = 20 µm.

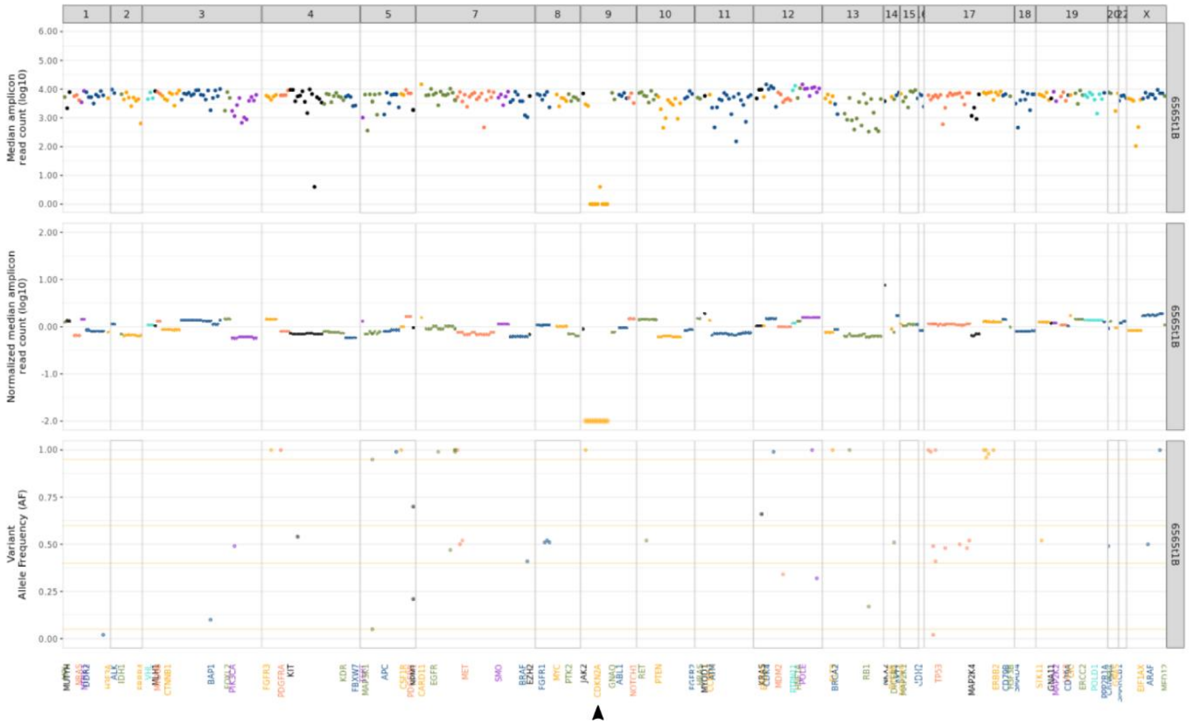

Supplementary Figure 4. Copy number variation analysis based on cancer hot spot panel sequencing of L6565. Upper panel: logarithmic scale, each dot represents the median read count per amplicon. Middle panel: normalized read counts. Lower panel: variant allele frequency. Arrow indicates the copy number loss of CDKN2A.

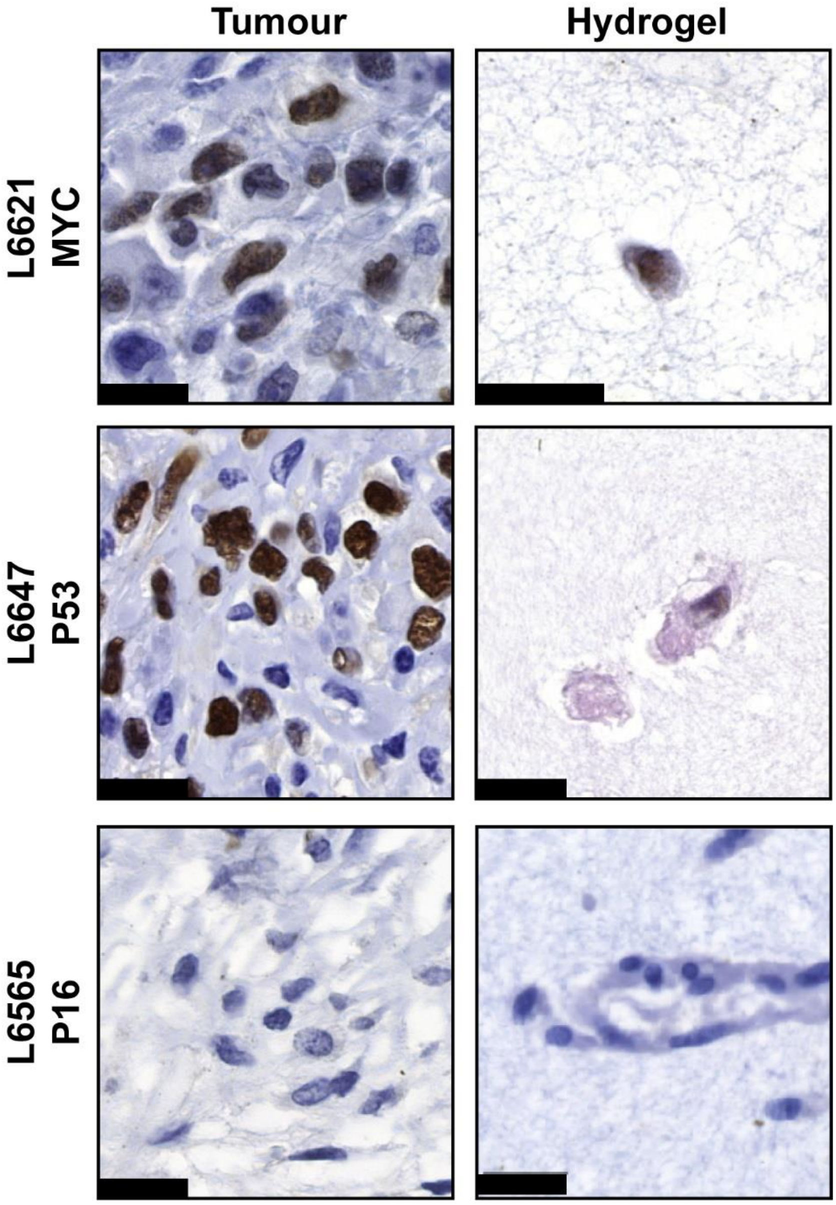

Supplementary Figure 5. Osteosarcoma hydrogels carry the same alteration as the original tumour. Loss of immunohistochemical expression of P16, or overexpression of MYC and P53 were confirmed in the osteosarcoma hydrogels derived from L6565, L6621 and L6647 respectively. Scalebar represents 20 µm.

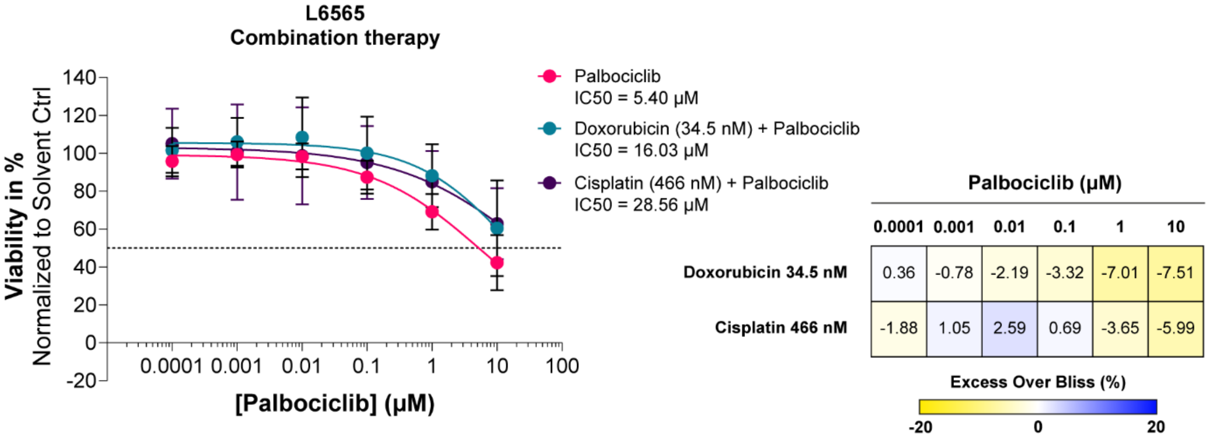

Supplementary Figure 6. L6565 2D cultured cells were treated with palbociclib combined with doxorubicin or cisplatin. The x-axis depicts the concentration of palbociclib. Cells were pre-treated with doxorubicin (34.5 nM) and cisplatin (466 nM) for 24 hours, after which palbociclib was added for 72 hours in total. The graph represents the average of four experiments performed In triplicate, with the standard deviation. A heatmap depicts the obtained Excess Over Bliss values, indicating combination therapy synergy, in which a value below zero represents antagonism (yellow), a value of zero represents additivity (white), and a value above zero represents synergy (blue).

Supplementary Table 1. Predefined list of targetable genes.

ABL1	CCND3	EPHA7	GNA11	MAP2K2	PALB2	SDHB
ABL2	CCNE1	EPHB1	GNAQ	MAP2K4	PAX5	SETBP1
ACVR1B	CD274	EPOR	GNAS	MAP3K1	PBRM1	SETD2
AKT1	CD79A	ERBB2	GRIN2A	MAP3K7	PDCD1	SF3B1
AKT2	CD79B	ERBB3	GRM3	MAPK1	PDCD1LG2	SMAD2
AKT3	CDH1	ERBB4	GSK3B	MCL1	PDGFB	SMAD3
ALK	CDK12	ERCC1	HGF	MDM2	PDGFRA	SMAD4
APC	CDK4	ERCC2	HNF1A	MDM4	PDGFRB	SMARCA4
AR	CDK6	ERG	HRAS	MET	PDK1	SMARCB1
ARAF	CDK8	ESR1	HSP90AA1	MITF	PIK3CA	SMO
ARID1A	CDKN1A	ETV1	HSP90AB1	MLH1	PIK3CB	SOCS1
ASXL1	CDKN1B	ETV6	IDH1	MPL	PIK3CG	SOX10
ATM	CDKN2A	EZH2	IDH2	MSH2	PIK3R1	SRC
ATR	CDKN2B	FANCA	IGF1R	MSH3	PIK3R2	STAG2
ATRX	CDKN2C	FANCC	IGF2	MSH6	PIM1	STAT3
AURKA	CEBPA	FBXW7	IKBKE	MTOR	PMS2	STAT4
AURKB	CHEK1	FGF23	IKZF1	MYB	POLD1	STAT6
AXL	CHEK2	FGF3	IL7R	MYC	POLE	STK11
B2M	CIC	FGF4	INHBA	MYCN	PRKDC	SUFU
BAP1	COL1A1	FGF6	IRS2	MYD88	PTCH1	SYK
BCL2	CREBBP	FGFR1	JAK1	NBN	PTEN	TERT
BCL2L1	CSF1R	FGFR2	JAK2	NF1	PTPN11	TET2
BCL2L2	CSF3R	FGFR3	JAK3	NF2	PTPRD	TGFBR2
BCL6	CTNNB1	FGFR4	JUN	NFE2L2	RAC1	TMPRSS2
BCOR	CXCR4	FH	KDM5A	NFKB2	RAD21	TOP1
BCORL1	DDIT3	FHIT	KDM5C	NOTCH1	RAD50	TOP2A
BLM	DDR2	FLCN	KDM6A	NOTCH2	RAD51	TP53
BRAF	DNMT1	FLT1	KDR	NOTCH3	RAF1	TSC1
BRCA1	DNMT3A	FLT3	KEAP1	NOTCH4	RARA	TSC2
BRCA2	DOT1L	FLT4	KIT	NPM1	RB1	TSHR
BRD4	EED	FOXL2	KMT2A	NRAS	RET	TYK2
BTK	EGFR	FOXO1	KMT2D	NTRK1	RICTOR	U2AF1
CALR	EP300	FUS	KRAS	NTRK2	RIT1	VEGFA
CBL	EPCAM	GATA1	LRP1B	NTRK3	ROS1	VHL
CCND1	EPHA3	GATA2	LRRK2	PAK1	RPTOR	WT1
CCND2	EPHA5	GLI1	MAP2K1	PAK3	RUNX1	XPO1

Supplementary Table S2. Targetable genes selected for seven osteosarcoma patient samples.

Patient ID	SNV, INDELS	CNV
**L6558**	n.a.	n.a.
**L6565**	ABL1; HNF1A; KMT2D; KRAS; PIK3CG; STAT3	CDKN2A (-)
**L6581**	DDR2	ERG (+), U2AF1 (-)
**L6620**	n.a.	n.a.
**L6621**	SETD2	ERG (+), MYC (+), IRS2 (+)
**L6647**	KMT2D; TP53	ATRX (+), KIT (+), KDR (+), PDGFRA (+)
**L6727**	MYB; RB1; TET2; TP53	n.a.
